# Trustworthy AI in Telehealth: Navigating Challenges, Ethical Considerations, and Future Opportunities for Equitable Healthcare Delivery

**DOI:** 10.1049/htl2.70020

**Published:** 2025-10-01

**Authors:** Seyyed Ali Zendehbad, Jamal Ghasemi, Farid Samsami Khodadad

**Affiliations:** ^1^ Faculty of Engineering and Technology University of Mazandaran Babolsar Iran; ^2^ Faculty of Engineering Modern Technologies Amol University of Special Modern Technologies Amol Iran

**Keywords:** BioMEMS, clinical decision support, LLMs, remote monitoring, smart wearable, telehealth, TAI

## Abstract

Trustworthy artificial intelligence (TAI) will transform telehealth by providing safe, transparent, and ethically compliant systems that enhance clinician decision‐making and patient relationships. This systematic review examines how TAI and large language models (LLMs), including large language model meta ai (LLaMA), can be integrated into telehealth systems, their role in optimizing e‐consultation workflows, and their capacity to support personalized care through data collected by wearable biosensors and biological microelectromechanical systems (BioMEMS). These devices monitor physiological and behavioral data, such as heart rate, blood pressure, and emotional state. TAI enables effective diagnostics and targeted treatment by combining various information sources, including biosensor readings, patient history, and cognitive data. Firmware integrity plays a crucial role in ensuring security, reliability, and continuous data encryption. This review analyses 135 papers (October 2020‐March 2025) from databases like IEEE Xplore, PubMed, and Scopus to demonstrate TAI's potential to enhance resource use and patient engagement. However, widespread adoption depends on overcoming technical challenges, improving firmware reliability, strengthening data security, and addressing ethical concerns. This review offers valuable guidance for engineers, system architects, and healthcare providers to create a sensitive and effective telehealth ecosystem.

## Graphical abstract

The graphical abstract illustrates how TAI telehealth systems integrate biosensors with knowledge‐based systems through LLMs and firmware. The diagram shows the continuous process starting with real‐time data collection and ending with secure, personalized healthcare delivery. The role of firmware is crucial in protecting data integrity and enhancing security measures, while remaining adaptable to changing telehealth needs.

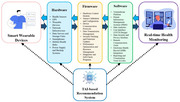

**Abbreviations**
Acronym: Full Form
AI: Artificial Intelligence
BioMEMS: Biological Microelectromechanical Systems
BLE: Bluetooth Low Energy
DL: Deep Learning
EHR: Electronic Health Records
GDPR: General Data Protection Regulation
HIPAA: Health Insurance Portability and Accountability Act
IMU: Inertial Measurement Unit
IoT: Internet of Things
KBES: Knowledge‐Based Expert System
LIME: Local Interpretable Model‐agnostic Explanations
LLaMA: Large Language Model Meta AI
LLMs: Large Language Models
LOC: Lab‐on‐a‐Chip
NLP: Natural Language Processing
PPG: Photoplethysmography
SHAP: SHapley Additive exPlanations
TAI: Trustworthy Artificial Intelligence
XAI: Explainable Artificial Intelligence

## 1 Introduction

**FIGURE 1:**
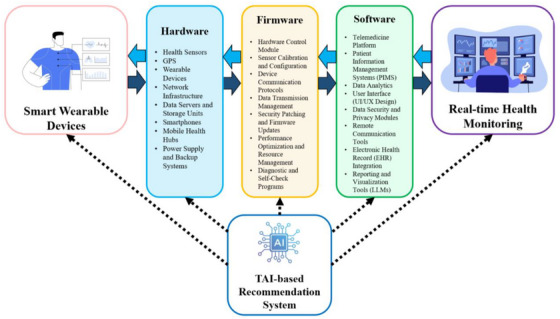
The schematic diagram presents a TAI‐based telehealth system integration that unites wearable biosensors with LLMs, knowledge‐based systems, and firmware. The displayed diagram shows how real‐time data acquisition progresses into processing and interpretation steps, which lead to secure, personalized health services. The system design clearly shows that firmware plays an essential function in protecting data integrity while providing security features alongside flexibility for changing telehealth needs [51].

TAI recognizes itself through transparency‐based principles and combines reliability, ethical accountability, and fairness while maintaining transparency and seeking to maximize these aspects for high‐risk healthcare environments [1]. The main goal of general AI involves maximizing performance metrics, but TAI places user trust and ethical requirements at its core [2]. Through TAI, telehealth maintains secure AI‐driven decisions along with full transparency and is free from bias to resolve privacy, consent, and bias‐related concerns [3]. The TAI frameworks integrate explainable AI (XAI) capabilities to demonstrate decision processes to health practitioners and patients, which builds their trust in the system [4]. The lack of safety mechanisms within general AI systems makes these systems prone to unexplainable decisions along with ethical complexities [5]. The healthcare industry is transforming thanks to TAI because this system offers secure, transparent, ethical solutions based on AI. The author emphasizes the critical role that TAI should play in sensitive domains, including healthcare, as well as finance and public policy. TAI enables free decision‐making together with protected, sensitive data management and transparent outcomes [6, 7]. The essential features of TAI rely on creating trust through bias reduction and explaining processes alongside delivering dependable and interpretable outputs [8]. TAI provides essential accountability features that make it a suitable choice for applications that need data security, interpretability, and data integrity, such as telehealth solutions [9, 10]. Healthcare business models, together with clinical decision support systems may experience transformation through the integration of TAI services, which include LLMs and LLaMA [11]. These models are advanced enough to generate human‐like text, engage in natural conversations, and provide decision‐making support in telehealth, a method of remote healthcare delivery through electronic means [12]. LMs can help improve e‐consultations and reduce communication barriers due to low health literacy and language differences through their functioning as clinician‐AI assistants and chatbots to optimize patient care delivery and streamline operational workflows [13]. The models function as virtual aids for e‐consultations while they interpret symptoms and assist with decision support. The LLaMA system stands out through its ability to merge medical field knowledge, which helps enhance care procedures and solve issues from inadequate health literacy and language mismatching problems [14]. Real‐time healthcare assistance enables better patient involvement since LLMs deliver medication prompters combined with health instruction, which helps patients follow their treatment plans and achieve superior outcomes [15]. The combination of data automation, summary creation, and instant assistance from LLMs decreases healthcare provider strain while creating a telehealth experience that focuses on patients. Through their capability to explain medical information, telehealth services become easier to use across different population groups [16]. However, essential areas of constraint remain; unlike the absence of access to appropriate AI regulations, privacy issue, and the ethicality of the application [17]. This study presents the first scoping review of LLM application research, including LLaMA, in telehealth, conducting a systematic analysis of existing research and identifying knowledge gaps [18]. The ethical ramifications of TAI will be the center of future research; the principles of transparency and explainability should be the focus of future research [19, 20]. Responsible LLM adoption in telehealth can lift the service quality and enhance the trust of patients and doctors so that sustained patient outcomes can emerge [21]. As well as the knowledge‐based expert system (KBES), which is also an AI‐based system that manipulates structured domain‐specific information, including medical guidelines and clinical protocols, together with expert rules for delivering support decisions [22]. Patient data analysis with evidence‐based recommendation generation happens through a combination of ontology systems and inference engines operating alongside rule‐based reasoning. Telehealth applications gain clinical decision power through KBES because they connect patient information to medical standards, which results in precise individualized treatment delivery [23]. The development of wearable and implantable biosensors that can monitor a wide range of physiological and behavioral metrics has enabled the adoption of such biosensors for remote monitoring of health, increasing the depth and accuracy of health remote monitoring [24]. However, today we now have the means to develop advanced wearable biosensors that can be integrated into smartwatches, rings, tattoos, patches, clothing, bands, and implantable devices to measure heart rate variability (HRV), blood oxygen saturation (SpO2), blood sugar, blood pressure, respiration rates, and drowsiness [25, 26]. Even these devices can measure electrolyte levels via sweat analysis [24]. They enable accurate and continuous monitoring of health metrics that are critical for real‐time management of stress, hydration, and metabolic states [27]. Furthermore, the emerging biosensors can record long‐term, and high‐accuracy data on peripheral nervous system information, fatigue, cognitive, and emotional states [28]. This capability enables healthcare providers to track patients' psychological and cognitive status and improve both acute and long‐term health outcomes [29]. Similar studies indicate that the effective use of TAI in telehealth relies on achieving a balance between high performance, reliability, stability, and security [30]. For an AI system to be deemed reliable in healthcare, it must perform consistently well in complex and noisy environments while also adhering to stringent standards of data integrity, privacy protection, ethical transparency, and overall reliability [31]. Moreover, the explainability of AI models is crucial, as it enables healthcare providers and patients to understand TAI‐supported recommendations [32]. This is particularly important in the design of critical, patient‐specific scenarios where rapid and accurate decision‐making is essential [33]. The growing body of literature on TAI for telehealth has underscored the capacity of knowledge‐based systems to aggregate complex, multi‐source data for deeper insights [34]. Studies emphasize the importance of integrating diverse datasets, including metrics from wearable biosensors, historical patient data, biopatterns, and contextual environmental factors [35]. This comprehensive data integration enables AI systems to identify subtle health trends, support preventive care, and, most importantly, provide actionable insights that respond to real‐time changes in a patient's health status [36, 37]. Furthermore, recent findings suggest that the active use of biosensors in telemonitoring significantly enhances patient engagement and outcomes, promoting a shift toward more preventive and patient‐centered healthcare models [38]. Despite significant advancements in hardware and software, firmware development remains a serious challenge in the operational implementation of AI systems in healthcare, including telehealth [39, 40]. Firmware acts as an intermediary between hardware and software, requiring continuous updates and optimization to adapt to the rapid changes in technology and the complex needs of operational environments [41]. Incompatibility or weaknesses in firmware development can lead to issues in the performance, security, and overall reliability of AI systems, which is particularly critical in situations where rapid and accurate decision‐making is essential for patient health. Therefore, the challenges associated with firmware development require special attention to effectively harness the potential of AI in improving healthcare service quality [42]. Firmware is important to the effective operation of AI systems in telehealth as it controls and manages the performance of complex hardware components [43]. At the low level, these software programs allow systems to collect and analyse multi‐source data, for example, from wearable biosensors, historical patient data, or biological patterns [44]. In addition to helping detect subtle health trends and supporting preventive care, this capability also provides actionable information to help respond to real‐time changes in patient health status [45]. Also, firmware is a security layer protecting the patient's sensitive information and keeping systems protected from threats and vulnerabilities [46]. As a result, the development and optimization of firmware are crucial for the development and optimization of reliable and effective AI systems in telehealth [47]. Figure 1 presents the core architecture of a TAI‐based telehealth system, as established through comprehensive studies and the authors' expertise in telehealth AI integration. This diagram depicts the critical elements needed to ensure robust telehealth services that can manage diverse clinical demands. These components address core functions in real‐time data acquisition, processing, and interpretation, integrating biosensor data streams, patient historical data, and contextual health information. Each module contributes to creating a seamless pathway for secure data transfer, real‐time processing, and personalized decision support [48]. The review evaluates quick advancements in AI‐based telehealth to explain how TAI, alongside knowledge‐based systems, creates distant health monitoring systems and medical decision platforms that improve individualized care delivery via enhanced healthcare provider‐patient data interaction. Healthcare providers obtain real‐time patient‐tailored care through these systems while resourcefully interpreting patient healthcare information. The analysis identifies essential barriers to telehealth reliability that involve protecting data privacy, ensuring system interoperability, and integrating data from multiple sources [49]. This review investigates healthcare challenges to assist engineers and system architects in developing secure telehealth platforms that unite AI innovations with healthcare requirements for quality and accessibility. In addition, organizations must follow standards such as the Health Insurance Portability and Accountability Act (HIPAA) and Food and Drug Administration (FDA) regulations to achieve safe and proper deployment of telehealth systems driven by AI [50]. To make key concepts easier to understand, Table 1 sorts TAI components by clinical, technical, and regulatory factors to address the gaps noticed in the literature.

**TABLE 1:** Taxonomy of TAI Components in telehealth.

Component	Subcategories	Clinical relevance	Key challenges
Transparency	XAI Auditability	Builds clinician trust Required for critical decisions	Computational overhead
Security	Data Encryption Firmware protection Network security	HIPAA/GDPR compliance Prevents breaches	Latency in encrypted data
Fairness	Bias mitigation Equity assurance	Prevents care disparities Inclusive diagnostics	Minority group underrepresentation
Reliability	Noise robustness Performance consistency	Critical for emergency monitoring (e.g., arrhythmia)	Power consumption in wearables

Finally, our research question investigates how TAI technologies affect telehealth platforms in terms of technical aspects and ethics, alongside operational effectiveness, and their comparison with conventional AI‐based telehealth systems regarding clinical results, system performance, data protection, and ethical compliance. The article brings the following main contributions to the area of trustworthy AI in Telehealth:

- A review of 135 studies (2020‐2025) that examined the difficulties in using TAI in telehealth, focusing on technical, ethical, and regulatory issues.
- A new structure using LLMs (e.g., LLaMA), BioMEMS, and improved firmware to enhance the security and fast operation of telehealth systems.
- Examining how wearable and implantable biosensor data can be used with AI to give personalized recommendations.
- Reviewed LLM applications in the context of telehealth, discovering possible issues (for example, hallucinations) and ways to manage them.
- The ability to develop firmware and security protocols practically for AI‐driven telehealth devices.

The following section (Section 2) discusses the methodology and how the research was conducted. Section 3 covers the telehealth method, involving hardware, firmware, and devices worn by patients. Section 4 discusses how AI affects the accuracy of diagnosis and treatment for patients. Section 5 examines LLMs and knowledge‐based systems employed in clinical decision support. Section 6 of the chapter looks at BioMEMS being used in remote monitoring. Section 7 deals with technical issues, the risk of unauthorized access, and moral issues. Finally, Section 8 highlights significant results and discusses what can be done in the future.

## 2 Method and Materials

### 2.1 Database Selection Rationale

Our research objectives required a systematic review of articles between October 2020 and March 2025 from major databases that specialize in AI, telehealth, and healthcare fields. Our research utilized the databases IEEE Xplore and PubMed, together with Scopus, ScienceDirect, and Google Scholar. IEEE Xplore served as the top priority database because it contains a vast collection of peer‐reviewed articles and conference papers focused on AI and engineering applications in healthcare, which provide essential resources for telehealth technical advancement. The extensive biomedical content available through PubMed made it an ideal choice because it enabled researchers to access telehealth studies focusing on clinical practice and patient results. The research utilized Scopus and ScienceDirect because these databases offer multidisciplinary research access to high‐quality studies from engineering, computer science, and medical sciences fields. The research included Google Scholar because it allowed the retrieval of scholarly articles from diverse sources, which encompassed materials not typically found in conventional databases. The authors chose these databases due to their expertise in building a solid foundation for identifying high‐quality research on TAI and telehealth applications. The three databases provide comprehensive scholarly coverage of technical advancements, alongside clinical studies and multidisciplinary research, which fulfill the study requirements.

### 2.2 Literature Selection Rationale

**FIGURE 2:**
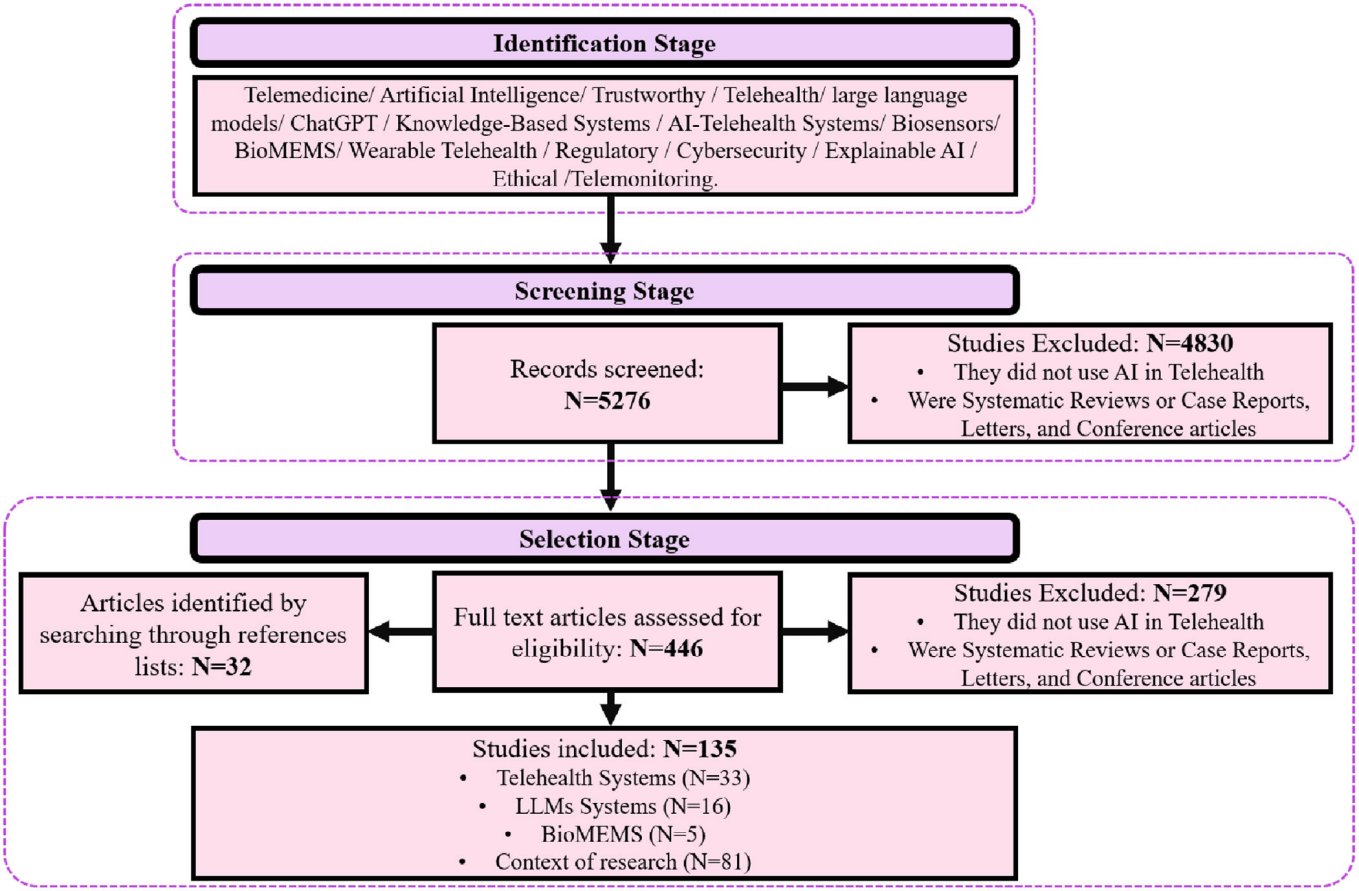
The PRISMA flow diagram of the literature search.

To include a broad range of relevant keywords, we used Boolean logic in crafting our search strategy, including: ‘Telemedicine’, ‘AI’, ‘Trustworthy’, ‘Telehealth’, ‘LMM’, ‘ChatGPT’, ‘Knowledge‐Based Systems’, ‘AI Telehealth Systems’, ‘Biosensors’, ‘BioMEMS’, ‘Wearable Telehealth Systems’, ‘Regulatory’, ‘Cybersecurity’, ‘Explainable AI’, ‘Ethical’, and ‘Telemonitoring’. These terms were combined with operators such as ‘AND’ and ‘OR’ to cover all literature relevant to AI applications in telemedicine and telehealth systems. Only articles that met strict criteria were included: articles that used AI models in telemedicine, telehealth, or recommendation systems for wearable health monitoring in original studies. Excluded were reviews, letters, conference papers, case reports, and studies not falling within the specified criterion. The final sample of 135 articles reflects a refined approach to this topic, shedding light on AI‐driven progress in telehealth. The PRISMA‐style flow diagram is presented in Figure 2.

### 2.3 Inclusion and Exclusion Criteria

The systematic review included strict selection standards, which enabled researchers to pick 135 methodologically sound articles from a large pool of studies. The study selection focused on original research articles published in English that examined TAI in telehealth across remote patient monitoring, clinical decision support, and patient engagement systems from October 2020 through March 2025. The review excluded non‐empirical research types, including reviews, letters, editorials, conference papers, and case reports, together with research on unrelated subjects or non‐English publications, to maintain focus on novel and empirical evidence within specified boundaries.

### 2.4 Data Extraction Paradigm

In this phase, we describe the structured approach to extracting, evaluating, and synthesizing data from relevant studies, with a focus on AI applications in telehealth. 135 primary sources were reviewed thoroughly in Figure 2 for comprehensive data extraction, based on publication year, citations, and research focus. Analysis Techniques: This review identifies how AI improves telehealth by identifying methods in telemedicine applications, from extracting features in signal processing to machine learning (ML) models used for clinical decision‐making. We examined techniques such as natural language processing for patient interaction, predictive modeling for chronic disease management, and deep learning (DL) in biological signal processing to understand their impact on remote patient care.

- Empirical evidence: To extract substantial evidence, four core components were reviewed.
- Datasets utilized: Most studies used public and proprietary datasets of biometric, behavioral, and health metrics (e.g., Electroencephalogram (EEG), Electrocardiogram (ECG), PPG data).
- Features for analysis: The extracted features included HRV, oxygen saturation, cognitive load indicators, and emotional states, which are essential to assess patient health in real time.
- Models/methods applied: The methodologies were supervised and unsupervised learning, expert systems, and knowledge‐based AI for problem domains of early stress and fatigue detection, symptom prediction, and patient engagement.
- Evaluation metrics: Novelty, Accuracy, specificity, sensitivity, and latency were standard metrics used to assess model performance, which reflect the system's reliability in real‐time, life‐critical applications.

**FIGURE 3:**
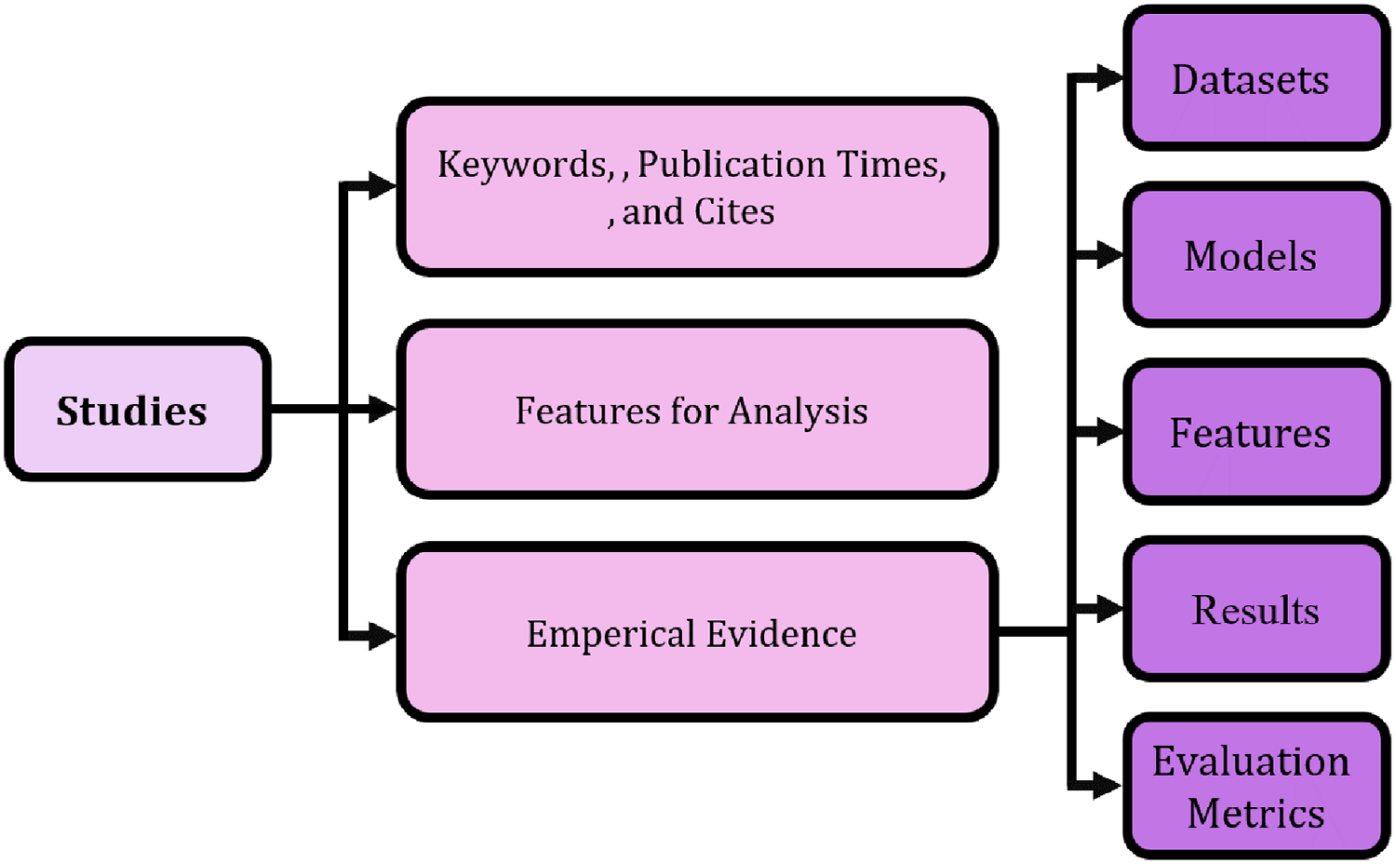
A summary of the systematic review data includes essential features found in the included research studies. The visual depicts the frequencies of AI systems, including LLMs, DL, and knowledge‐based systems, as well as telehealth solutions, starting from remote monitoring, e‐consultations, and diagnostic imaging, with evaluation criteria such as accuracy, sensitivity, and latency.

This systematic extraction and monitoring process offers a data‐driven path for designing telehealth systems to optimize patient outcomes and operational efficiency, and a foundation for addressing our research goals. Figure 3 shows the overview of the extracted data.

## 3 Telehealth Paradigm in the Health Care System

With the advent of hardware, firmware, smart wearable devices, and TAI‐based recommendation systems, telehealth has changed the patient monitoring and treatment modality. It has been integrated into the healthcare system [52]. Wearable biosensors, together with electronic health records (EHRs), environmental sensors, and patient‐reported outcomes, need to be unified through a framework for analytical purposes. The combination process of diverse data sources includes data preprocessing, followed by normalization and the use of fusion techniques to achieve consistency and accuracy standards [53]. Telehealth benefits from multi‐source data integration, which creates extensive patient surveillance systems, time‐sensitive clinical guidance, and individualized treatments that result from complete patient health information analysis [54]. In this section, each component shown in the telehealth system model is dissected and its role and interactions in building a smooth and patient‐centric remote healthcare environment are described.

### 3.1 Smart Wearable Devices in Telehealth Systems

**FIGURE 4:**
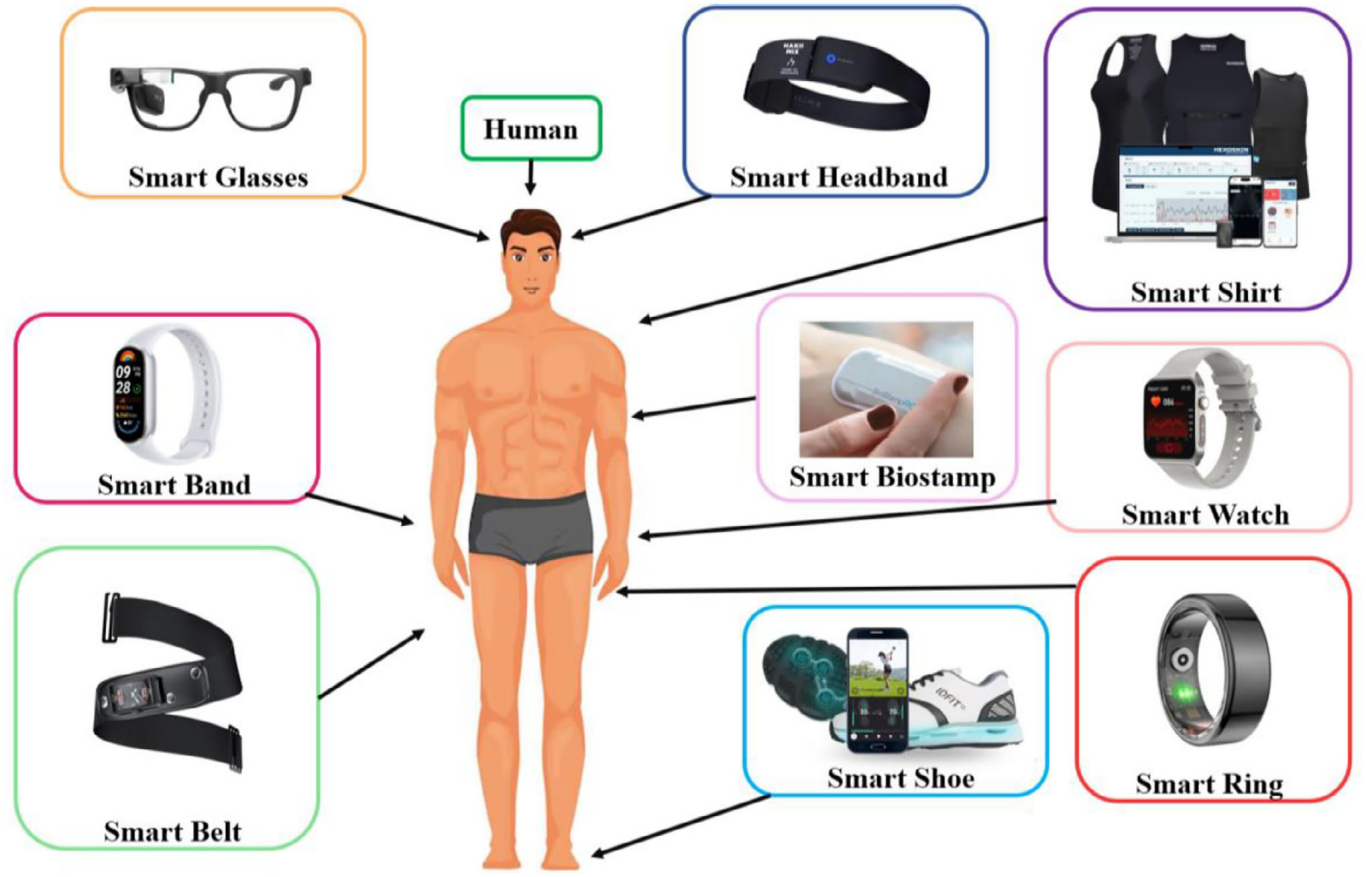
The various types of smart wearable devices for telehealth applications.

Telehealth is built on smart wearable devices that continuously and non‐invasively monitor key physiological metrics [55]. As shown in Figure 4, some examples include smartwatches, bands, rings, shoes, glasses, shirts, etc. [56, 57, 58, 59, 60, 61, 62]. Essential health data such as heart rate, SpO2, blood pressure, respiratory rate, glucose level, and electrolyte balance are collected by these devices through sweat analysis [63]. Also, they can track fatigue, sleep quality, cognitive performance, and emotional states for complete health tracking [64]. Knowing this information helps in early identification of health issues and managing chronic problems. Wearables are a reliable source of real‐time health data because of their adaptability and ease of integration into daily life [65]. There are wearable devices that use different sensor technologies to monitor key physiological metrics. Photoplethysmography (PPG) is an optical sensor technology that is mainly used to measure blood flow and heart rate [66]. It works by pumping light in the green or infrared range, typically pumped into the skin, and the reflected light is measured [67]. The detection of heart rate and SpO2 levels is enabled by variations in light absorption caused by pulsatile blood flow in the microvascular bed of tissues [68]. It's commonly included in smartwatches and fitness trackers. Inertial measurement units (IMU) contain accelerometers and gyroscopes that, when combined, detect motion and orientation changes [69]. Tracking physical activity, detecting falls, and gait patterns require these sensors [70]. IMUs in wearables are also helpful for monitoring user movements and activity levels, as well as fatigue and physical performance [71]. Temperature sensors monitor the body or skin temperature of the wearer to monitor metabolic activity and fever and indicate general health status [72]. Thermistors or infrared technology can be used by these sensors, which are usually integrated into smartwatches or health monitoring patches [73]. Furthermore, the Apple Watch includes PPG sensors that serve multiple functions, including constant heart rate tracking and atrial fibrillation (AFib) detection. The study performed large‐scale research, which demonstrated that the Apple Watch maintained high precision in detecting irregular heart patterns, thus providing timely medical intervention to at‐risk populations [74]. On the other hand, the Dexcom G6 and Abbott FreeStyle Libre, along with other continuous glucose monitoring (CGM) systems from Dexcom, have transformed diabetes management through their capabilities to show immediate glucose levels and patterns. Remote patient monitoring through wearable devices helps telehealth programs track diabetic patients' health status without requiring regular clinic appointments, which enhances their glucose control [75]. The AliveCor KardiaMobile serves as a portable ECG monitor that telehealth platforms utilize for remote diagnosis of cardiac arrhythmias [76]. Also, the Medtronic Reveal LINQ represents one of many implantable biosensors that prove effective at extended cardiac monitoring for patients with unexplained syncope or arrhythmias. The devices send data through wireless networks to telehealth systems, which provide ongoing observation and instant medical care during detected irregularities [77]. The emerging lab‐on‐a‐chip (LOC) technology system revolutionizes remote diagnosis through miniature laboratory equipment, which enables sophisticated biochemical tests (such as blood and urine evaluation) to be executed outside standard laboratories. The functionality proves essential for locations with restricted access to diagnostic centers [78]. These sensor technologies are used to improve telehealth and enhance how wearable devices can provide comprehensive health monitoring, benefiting patients and improving outcomes. Table 2 categorizes various biosensor technologies based on their primary features.

**TABLE 2:** Taxonomy of telehealth biosensor technologies.

Sensor Type	Measured parameters	Accuracy	Latency	Clinical applications	Key limitations
PPG	Heart rate (HR) SpO2	±2 bpm ±1% SpO2	< 1 s	AFib detection	Motion artifact susceptibility
ECG	Cardiac rhythms ST segments	99.5% sensitivity	5 s	Post‐op monitoring	Limited high‐frequency resolution
CGM	Blood glucose	MARD 9.2%	5 min	Diabetes management	Calibration drift over time
IMU	Gait patterns Falls	95% sensitivity	100 ms	Elderly care	Orientation‐dependent errors
Thermal	Skin/core temperature	±0.1°C	30 s	Fever monitoring	Ambient temperature interference
EEG	Neural oscillations Cognitive load	±5 µV resolution	50 ms	Seizure detection Mental state assessment	High sensitivity to noise
EDA	Skin conductance Stress response	0.01 µS resolution	2s	Emotional state tracking Pain monitoring	Sweat/skin condition dependence

### 3.2 Hardware in Telehealth System

A telehealth system consists of hardware that includes the sensors and modules needed for data acquisition and transmission [79]. Global positioning system (GPS), wearable sensors (biometrics) such as PPG, network modules (communication), storage units (data retention), data transfer hubs, and display systems are components [80, 81]. Together, these elements make for an easy way to capture and relay data [82]. In particular, network modules and data transfer hubs are unique in transmitting patient data to the central systems, and storage units provide a safe place to store patient information for future analysis [83]. Accordingly, the display systems demonstrate visual feedback to healthcare providers for timely interventions [84].

### 3.3 Firmware in Telehealth System

The operational backbone is firmware, which controls hardware performance, data processing, and security protocols. Sensor calibration and configuration, data encryption for privacy protection, cyber threat protection, performance tuning, and system updates are provided by key functions [51]. Efficient data transmission from the sensors to central servers is achieved with the help of advanced firmware, while data integrity is preserved [85]. The firmware enables adaptability to new telehealth requirements and optimizes wearable device function in the healthcare infrastructure by allowing regular updates and diagnostics [86].

### 3.4 Software in Telehealth Systems

A telemedicine platform, patient management systems (PMS), data analytics, and user interface (UI) modules are the software layer of the system which are critical to the system's usability and effectiveness [87]. Telemedicine platforms offer virtual consultations, and PMS organizes patient data for easier access by healthcare providers [88]. AI‐enabled data analytics modules process and interpret health data to come up with patient conditions that they can feed to doctors [89]. Using blockchain technology and a cloud service, these tools of novel communications enable effective patient healthcare interactions [90, 91, 92]. They also assist software tools by making complex data easier to understand and facilitating data‐driven applications. EHR integration also plays out in enabling the sharing of patient data between healthcare systems for continuity of care and eventually increasing patient outcomes [93].

### 3.5 Real‐Time Health Monitoring

For telehealth, real‐time health monitoring is crucial to provide real‐time insights into patients’ health metrics [94]. Wearable sensors provide the data, which are processed by hardware and managed by firmware to be transmitted to healthcare professionals. Being able to monitor in real‐time means getting proactive about trends seen, for example, abnormal heart rates or blood pressure spikes [95]. This dynamic monitoring capability supports preventive healthcare by improving patient outcomes through more action at the time of early signs of health deterioration, allowing clinicians to act on early signs and adjust treatment as necessary [96]. The effectiveness of smart wearable systems in telehealth relies heavily on specific communication protocols that support reliable, rapid, and stable data transmission [97]. In the proposed system, several communication protocols are suggested to establish secure links among sensors, wearable devices, mobile phones, satellites, and servers, alongside blockchain and a cloud service solutions to ensure safe and accessible data handling [98]. These protocols, commonly used in telemedicine and the internet of things (IoT), each offer distinct advantages and limitations [99]. For example, bluetooth low energy (BLE) is widely adopted for connecting wearables like smartwatches, fitness bands, and patches to mobile devices due to its low power consumption [100]. BLE enables real‐time transmission of biometric data (e.g., heart rate, respiratory rate) with minimal battery usage, making it ideal for compact devices [101]. However, with a limited range (up to 100 m), BLE is less suitable for long‐distance communication or satellite connectivity [102]. In contrast, Wi‐Fi provides higher data transfer speeds and a broader range than BLE, making it preferable for transmitting large datasets, such as ECG or EEG readings, from smart patches or clothing to cloud servers [103]. While effective for high‐volume data transfer, Wi‐Fi's high energy consumption poses challenges for battery‐constrained wearables [104]. Additionally, it relies on local network availability, limiting its use in remote areas [105]. Mobile networks (4G/5G) are particularly advantageous for wearables that require constant connectivity, such as SOS and GPS‐enabled devices. The high speed and low latency of 5G, in particular, allow for real‐time processing of biometric data, which is essential for emergency monitoring [106, 107]. However, mobile networks may have limited coverage in remote regions, and data costs are typically higher than BLE or Wi‐Fi [108]. Low earth orbit (LEO) satellites offer connectivity in areas lacking mobile network coverage, enabling remote monitoring with devices like Garmin inReach Mini for SOS messaging and GPS tracking [109]. Although satellite communication is effective in these settings, it is more costly, can experience delays, and generally requires devices with larger form factors and higher energy needs [110, 111]. Zigbee, a low‐power protocol designed for IoT devices, is suitable for smart patches and small biosensors that require low‐energy, short‐range data transmission across interconnected devices [112]. Its slower data rate, however, limits its use for high‐volume data applications such as EEG monitoring [113]. Also, LoRaWAN is optimal for long‐range, low‐power data transmission from biosensors in wide‐area networks, particularly suited for simple data like temperature, blood pressure, and heart rate [114]. Finally, to ensure secure data storage and accessibility, blockchain technology provides a decentralized, tamper‐proof record of data transactions, enabling traceability and security for patient data across the telehealth ecosystem [93, 115, 116]. However, due to its limited data rate, it is unsuitable for real‐time or complex data transfers (e.g., ECG and EEG). Each protocol presents distinct trade‐offs concerning range, energy consumption, and data transmission capacity, and the selection depends on the specific telehealth application and operational needs of the wearable device. To achieve optimal performance, the chosen protocol must meet essential parameters: reliability, stability, and availability across all conditions [117]. Healthcare providers can protect telehealth platforms through blockchain technology when they transmit real‐time data from wearable biosensors. Healthcare providers protect patient data integrity and confidentiality by using both encryption and blockchain‐based access event recording during data transmission [97]. By fusing data from medical devices attached to patients with EHR systems and environmental sensors, physicians can execute nonstop live healthcare observations that help promptly identify abnormal events and implement prompt responses [118].

### 3.6 AI‐Based Recommendation System

The intelligent core of telehealth is the TAI‐based recommendation system, which takes data and turns it into actionable insights. Multi‐source inputs, such as real‐time data from wearables, historical patient records, environmental factors, and biopatterns, are fed into advanced AI algorithms that recommend personalized healthcare [119]. These systems can identify subtle trends, help prevent problems, and help make clinical decisions. Explaining AI (XAI) models increases transparency to let healthcare providers and patients know exactly what these models suggest to them. In addition, the AI system's ability to learn and improve on different datasets makes it a key piece in achieving reliable and patient‐centered care [120].

## 4 The Role of AI in Telehealth

Its adoption into the field of telehealth has proven to be an integration of AI as a transformative agent that reshapes how healthcare services can be delivered remotely. There are many aspects of AI's role in telehealth, including diagnostic accuracy, personalizing treatment plans, resource allocation efficiency, and patient engagement. AI systems achieve improved telehealth efficiency through a combination analysis of various health data sources, which enables them to detect small medical patterns and forecast patient results as well as direct resources effectively. In this section, we will explain these aspects and how AI technologies will change the future of telehealth.

### 4.1 Deep Learning in Telehealth

Telehealth allows diagnostic imaging research and predictive modeling as well as natural language processing (NLP) sessions to progress through DL algorithms that rely on convolutional neural networks (CNNs), recurrent neural networks (RNNs), and attention‐based models. Research shows that CNNs deliver biomedical data analysis and pattern recognition results that equal or surpass human professional standards [34]. The clinical decision support system in telehealth now uses transformer‐based LLMs (LLaMA and ChatGPT) for human‐like interactions. Two fundamental challenges exist for DL algorithms because they require high‐quality datasets, but their black‐box operation does not satisfy the healthcare requirements of TAI [121]. However, the major applications of these algorithms involve the use of ML and DL to analyse significantly large volumes of data very accurately and very quickly [122]. In telehealth, AI can help healthcare providers diagnose conditions by looking at patient‐reported symptoms, medical history, and diagnostic imaging [123]. For example, AI‐based image recognition systems can read radiological images and identify anomalies with the same accuracy, which is sometimes better than that of human experts [124]. In particular, in remote settings in which there might be limited access to specialists, this capability is particularly crucial. Moreover, NLP technologies are designed to allow AI to interpret nonstructured data from clinical notes as well as patient interaction and pull out valuable insights to aid in diagnosis [125]. AI not only improves patient outcomes but also cuts down on the strain of the process on healthcare professionals by reducing the burden on them [126].

### 4.2 Personalizing Treatment Plans

Without AI, responsible for creating personalized treatment plans, telehealth services can't exist. AI algorithms can learn to recommend personalized therapy for several diseases using data from EHRs, wearables, and genetic information [127]. This is bringing precision medicine, where we treat a patients based on their characteristics and needs to achieve precision [128]. In addition, the computer can track patient progress sequentially and adjust treatment plans in real‐time [129]. For instance, ML models can also determine patient response to some therapies so that healthcare providers can decide when to adjust their treatment plans [130]. This dynamic patient personalization not only gains the patient's approval and improves adherence to treatment protocol, but it can also provide considerable benefits to the clinic [131]. The management of diabetes, together with hypertension and cardiovascular conditions, heavily depends on predictive modeling systems. Healthcare providers can use predictive models that combine EHRs and additional patient data sources, including biosensor data and genetic information, to forecast disease progression, identify high‐risk patients, and develop personalized treatment recommendations. Healthcare providers benefit from these models because they enable a transition to proactive care systems, which enhance patient wellness outcomes while lowering healthcare expenses [132]. Computer algorithms analysed real‐time glucose measurements to provide results about future blood sugar patterns in diabetic patients, according to the study by Udegbe et al., [127]. The article by Yogeshappa describes how predictive models utilize AI to optimize hypertensive patient treatment through personalized plans based on individual patient risk assessments [129]. The trial conducted by Ramírez et al. showed how machine learning algorithms succeeded in predicting diabetes treatment patient outcomes to achieve better glycemic control as well as minimize complications [130]. Predictive models used for chronic disease management encounter multiple obstacles when they are deployed for applications in disease supervision systems. Data accuracy, as well as algorithmic discrimination and routine model validation, needs resolution to guarantee these models operate correctly. Future research must concentrate on building predictive model resilience, as well as combining multiple data sources with ethical standards when using them in clinical environments [133].

### 4.3 Optimizing Resource Allocation

Telehealth system resource allocation can be more efficiently done by AI, which can analyse data patterns. Through predictive analytics, you can identify patient populations at risk for certain conditions, and healthcare systems can then proactively allocate resources, not reactively [134]. For instance, AI is capable of anticipating that there will be a spike in demand for telehealth services during epidemics, and hence, the provider should be prepared to change its staffing and equipment accordingly [135]. AI‐driven chatbots and virtual assistants can also triage patient inquiries so that health professionals don't have to spend time with trivial problems that require human intervention. Such optimization of resources is especially advantageous in telehealth, as the availability of well‐trained people and technology must be maintained at the highest level at times, and thus, this optimization is beneficial [136].

### 4.4 Methods to Improve Explainability in AI Decisions

The implementation of TAI in healthcare requires explainability to provide transparency, which enables trust‐building between healthcare providers and patients. The AI model interpretability is enhanced through the methods SHapley Additive exPlanations (SHAP) and local interpretable model‐agnostic explanations (LIME) [137]. SHAP calculates Shapley values for fair feature contribution assessment to support clinical decision systems that forecast cardiovascular risks and evaluate treatment outcomes [138]. Local predictions generated by LIME provide medical staff with explainable insights that enable customized treatment plans and diagnostic procedures. The two methods serve telehealth applications by offering explanations for AI‐driven decisions within chronic disease management and emergency care [139]. The system faces obstacles due to its complex computational operation or data quality issues. Researchers need to optimize the methods for AI integration and implement them in clinical workflows to build healthcare provider trust in AI systems [140].

### 4.5 Improving Patient Engagement in AI‐Driven Telehealth Systems

Telehealth systems that use AI require patient engagement to achieve their success goals. The evaluation of patient satisfaction scores, along with adherence rates and system usage frequencies, together with patient‐reported outcomes (PROs), enables healthcare professionals to measure successfully interactive system performance and improved health results [141]. The engagement from AI‐based recommendation systems functions through enhancing care recommendations specific to patients, along with real‐time support systems that adapt their communication method based on each patient's need [142]. Various research investigations have shown that AI technology enhances patient engagement practices. The use of AI‐powered telehealth platforms, as described in Lyles et al., led to better patient satisfaction and healthcare adherence since these systems deliver individualized and time‐sensitive health information [143]. AI plays an active role in improving patient engagement by combining interactive systems with time‐sensitive patient monitoring, according to Alowais et al. [136]. Furthermore, they are part of AI technologies in telehealth, offering interactive and user‐friendly interfaces. Chatbots and even virtual health assistants help patients feel less helpless. They make help available to them on demand for things like information, scheduling appointments, and medication reminders. AI can help personalize communication strategies for health information by producing this information in a personalized format to individual patient‐level preferences and literacy [144]. The measurement of patient engagement faces obstacles mainly because of difficulties in protecting patient data privacy, as well as developing universal performance indicators. Future investigation needs to resolve current barriers and create new solutions that will increase patient involvement in AI‐powered telehealth technology [145].

## 5 Telehealth, LLMs, and Knowledge‐Based Systems

Telehealth, LLMs, and knowledge‐based systems at the intersection are a game‐changing shift in how healthcare can be delivered and optimized remotely [146]. This paradigm leverages advanced AI frameworks (e.g., LLaMA, ChatGPT, Llama 2) to improve decision‐making, support real‐time patient interaction, and improve clinical outcomes through a continuous flow of information [147, 148]. In this blog, we explore each subcomponent of this integration and its telehealth implications [149]. LLMs in Telehealth LLMs and knowledge‐based systems represent a transformative shift in how healthcare can be delivered and optimized remotely [150]. By leveraging advanced AI frameworks, particularly models such as LLaMA, this paradigm aims to enhance decision‐making, support real‐time patient interaction, and improve clinical outcomes through a seamless flow of information [14]. Here, we will delve into each subcomponent of this integration and its implications for telehealth [151]. Furthermore, whether commercial or industrial products, blockchain technology, together with AI‐based recommendation systems, maintains data security and data traceability. Transparency plays a vital role in developing trust in AI‐based telehealth platforms because of their high‐risk clinical implementation [152]. Finally, Telehealth platforms that use LLMs need to fulfill all FDA medical device approval standards and HIPAA data privacy rules to provide safe patient care and maintain their trust [153].

### 5.1 The Role of LLMs in Telehealth

The use of LLMs, including most recently LLaMA, has dramatically changed telehealth by empowering sophisticated language processing and natural conversation abilities [154]. For telehealth, LLMs can work as clinicians' and patients' virtual assistants, bridging language barriers, interpreting the symptoms the patient described, and interpreting the medical information so that it can be easily understood [155]. This helps models to facilitate nuanced, context‐aware conversations that can span gaps in low literacy and language differences. In e‐consultations, LLMs can add to the clinical workflow by collecting patient information, generating summaries, and identifying key insights to streamline the clinical workflow and help to deplete the cognitive load of healthcare providers [156].

### 5.2 Knowledge‐Based Systems for Clinical Decision Support

Knowledge‐based systems in telehealth are used as a repository of medical knowledge, decision protocols, and clinical guidelines. By integrating with LLMs, Knowledge‐Based Systems improve the reliability and accuracy of AI‐driven recommendations using verified medical data and contextual patient information [157]. This integration allows the development of a robust clinical decision support system that provides diagnostic pathways, suggests treatment options, and offers evidence‐based advice for complex cases. LLaMA's adaptability to domain‐specific knowledge allows it to work concurrently with Knowledge‐Based Systems so that clinicians receive real‐time insights that are rooted in established medical practice and research [158]. The KBES system helps identify chronic diseases through its integration of fresh test results with past medical documents, as well as care standards [159].

### 5.3 Practical Risks of LLMs in Telehealth

The practical risks associated with LLMs in telehealth need careful management because they present substantial potential for telehealth improvement. The occurrence of ‘hallucinations’ stands as one of the significant risks because LLMs produce wrong and nonsensical information that misleads users. The combination of training data restrictions, ambiguous prompts, and probabilistic system operation leads to hallucinations. Healthcare facilities face the risk of inaccurate diagnoses, improper treatment decisions, and patient safety problems due to such system errors [160]. The distribution of unverified information presents a significant danger when LLMs are utilized. The responses created by LLMs might appear official but contain actual mistakes from old or biased information sources [161]. Accurate information plays a central role in telehealth, since patients and healthcare providers need reliable data to make their decisions. The incorrect delivery of medication dosage instructions and symptom misinterpretation by LLMs can generate unfavorable clinical outcomes that are potentially dangerous to patients [162]. Several measures should be implemented to reduce these risks. The integration between LLMs and knowledge‐based systems that maintain verified medical information to current standards should be the first approach. The implementation of evidence‐based practice systems alongside LLM helps to maintain factually correct responses. Second, human oversight is essential. Medical personnel need to examine LLM‐generated advice before taking any action. XAI techniques provide explanations about LLM decision‐making, which enables clinicians to both understand recommendation sources and detect potential mistakes [163]. The necessary step for LLM performance monitoring involves ongoing evaluation and detection of problems related to hallucinations and misinformation dissemination. Specified updates of training data must be combined with thorough clinical scenario testing, accompanied by feedback mechanisms that enhance model accuracy throughout its lifecycle. The integration of LLMs into telehealth systems becomes possible when practitioners handle these operational risks to achieve effective patient care with minimized adverse effects [164]. Table 3 outlines our risk taxonomy, which covers telehealth‐specific methods for validating prior LLM safety frameworks.

**TABLE 3:** Taxonomy of LLM risks and mitigation strategies.

Risk category	Manifestations	Mitigation strategies	Validation methods
Hallucinations	Factual inaccuracies Plausible but incorrect recommendations	Knowledge‐base grounding Human‐in‐the‐loop verification	Clinical trial testing Expert panel review
Bias	Demographic disparities Unequal performance across populations	Fairness‐aware fine‐tuning Diverse training datasets	Subgroup analysis Equity audits
Privacy	Data leakage risks Re‐identification potential	Differential privacy training Secure multi‐party computation	Penetration testing Compliance certification
Interpretability	Black‐box decision making Unclear reasoning chains	XAI techniques (LIME/SHAP) Decision provenance tracking	Clinician usability studies Transparency scoring

### 5.4 Scenario‐Based Applications and Use of LLaMA in Telehealth

**FIGURE 5:**
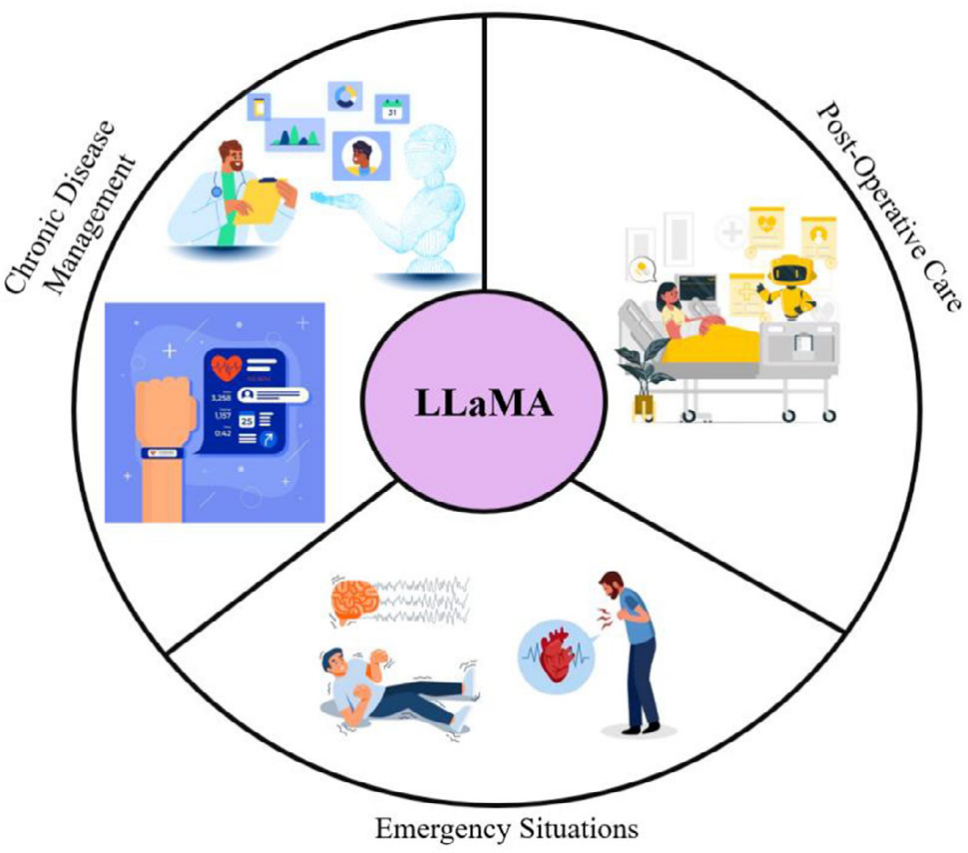
Illustrative applications of LLMs, such as LLaMA, in telehealth. The illustration shows three essential use cases where LLaMA combines chronic disease management, post‐operative care, and emergency response to boost healthcare decisions in regular and critical situations.

Moreover, LLaMA's potential in advanced, scenario‐based applications of LLaMA in telehealth is to tailor healthcare interventions to specific clinical needs. By simulating diverse patient scenarios, LLaMA can provide support to healthcare providers in being proactive with, and more personalized, care [149]. For example, in post‐operative monitoring, LLaMA can generate patient recovery trajectories so that providers can predict risks like infections or post‐surgical complications, and adjust care pathways accordingly. In chronic disease management, LLaMA can learn progression patterns of conditions like diabetes or hypertension, and generate patient‐specific insights and timely alerts for medication adjustments or lifestyle interventions [165]. In acute episodes (e.g., a sudden asthma attack or cardiac event), LLaMA's rapid data processing may support real‐time decision‐making and hence reduce critical response times [166]. In addition, LLaMA integrates biosensors worn by patients with historical patients and can further track the deviations of vital signs, giving early warnings for preventative care [167]. Some telehealth applications of LLaMA are visually represented in Figure 5, which shows that LLaMA can integrate real‐time patient data, environmental contexts, and disease models to improve clinical decision‐making in routine and emergencies.

#### 5.4.1 Chronic Disease Management

LLMs can manage patient history, medication adherence, and real‐time biosensor data such as a wearable blood glucose or blood pressure device. This can then provide personalized advice, recommend lifestyle adjustments, and inform patients or caregivers of possible health risks [168].

#### 5.4.2 Post‐Operative Care

LLMs can remind patients recovering from surgery about medication, assess their pain levels using conversation, and help patients take care of their wounds. The model reduces the likelihood of complications by proactively engaging the patient in adherence to post‐surgical protocols [169].

#### 5.4.3 Emergency Situations

In cases such as suspected heart attacks, epileptic seizures, or stroke, LLMs can very quickly evaluate symptoms reported by patients, risk factors, and can give quick instructions in the meantime until emergency services come. In these cases, LLMs’ speed and accuracy in processing symptoms and suggesting first‐response actions can be lifesaving [170]. Timely healthcare decisions in emergency care situations form the foundation for effective treatment because they directly enhance patient recovery. Healthcare staff now use a combination of LLMs and DL algorithms to accelerate their critical time‐based decision‐making through AI system support. Such systems analyse large volumes of information from wearable biosensors, besides electronic health records and real‐time patient inputs so that they can deliver valuable insights [171]. This technology enables stroke detection through examination of imaging systems and wearable device data, which helps medical professionals spot ischemic stroke in less than a minute. The research work by Ren et al. showcased a system with AI capabilities that monitors real‐time ECG data to predict cardiac arrest events and then warns medical personnel about necessary action to save lives [169]. The implementation of AI in trauma situations focuses on deciding patient treatment sequences by analyzing instant vital sign measurements and damage severity evaluations. Tabletop computers, along with deep learning models, support emergency decision‐making because they identify patterns in streaming medical data, and LLMs process complex healthcare information. Limitations remain in establishing reliable and accurate performance of these systems, especially during critical emergencies. Research studies should concentrate on strengthening AI systems' reliability while developing methods to implement them smoothly within current emergency medical system procedures [164]. Finally, Table 4 presents a clear summary that compares the key features, as well as benefits and drawbacks, of telehealth applications for AI frameworks analysed in this section.

**TABLE 4:** The comparison of different AI telehealth frameworks.

Framework	Key features	Strengths	Weaknesses	Applications
TAI	Transparency, explainability, ethical accountability, bias reduction.	Builds user trust, ensures ethical AI use, and reduces bias.	Requires high‐quality data and complex implementation.	Remote patient monitoring, clinical decision support, personalized treatment.
LLMs (e.g., LLaMA)	Natural language processing, human‐like interactions, and real‐time data analysis.	Enhances patient engagement, bridges language barriers, and improves workflows.	Black‐box nature, potential for bias, high computational cost, risk of hallucinations, and misinformation.	E‐consultations, symptom interpretation, patient education, virtual health assistants.
Knowledge‐based systems	Rule‐based reasoning, integration with medical guidelines, and structured data.	High reliability, evidence‐based recommendations, and domain‐specific knowledge.	Limited adaptability, requires frequent updates, and complex maintenance.	Chronic disease management, post‐operative care, and emergency response.

## 6 BioMEMS in Telehealth

BioMEMS are compact, integrated devices that combine biological elements with micro‐scale mechanical and electrical components, offering transformative potential in telehealth. Their small size enables minimally invasive monitoring, gathering continuous, high‐resolution data necessary for accurate health management in remote settings [172]. The high sensitivity and precision of BioMEMS make them vastly superior to legacy, larger sensors concerning the detection and subsequent monitoring of subtle physiological changes, which allow for greater accuracy of health monitoring and prompt interventions [173]. BioMEMS integrated with telehealth platforms enables seamless, real‐time data flow to healthcare providers who can use this information to make informed, immediate decisions that can help improve patient outcomes and reduce complication risks [174]. These systems also provide a cost‐saving solution in that they reduce the frequency of in‐person visits and eliminate the need for large‐scale medical equipment, making healthcare more accessible, particularly in underserved areas. In combination with AI, BioMEMS for telehealth creates personalized care and can provide treatments tailored to a patient's specific health profile. By integrating BioMEMS, telehealth, and AI, this integration of BioMEMS, telehealth, and AI provides a highly responsive, precise, and accessible approach to remote healthcare for patients and providers [175].

### 6.1 Implantable Biosensors in Telehealth

Embedded within the body, BioMEMS continuously monitors biometrics like blood glucose, respiratory rates, and other physiological parameters, offering real‐time data crucial for managing chronic conditions such as diabetes [176]. As well as providing a more reliable, stable measurement of internal biomarkers than external sensors, reducing the interference and signal artifacts often encountered in devices like PPG [177]. PPG sensors measure peripheral signals and can be affected by changes in ambient light, skin tone, and movement, which can degrade accuracy [178]. The medtronic reveal LINQ and other implantable loop recorders (ILRs) provide an established solution for long‐term cardiac monitoring, specifically in patients experiencing unexplained syncope or arrhythmias. The devices send information without wires to telehealth systems for continuous tracking and immediate medical assistance [179]. BioMEMS, embedded at the site of interest, offer a higher signal‐to‐noise ratio (SNR) and better resilience to such environmental factors, making them more robust and reliable for continuous patient monitoring [180].

### 6.2 Biosensor‐BioMEMS Integration in TAI Architectures

Using biosensors and BioMEMS in TAI systems provides a solid structure for safe remote healthcare monitoring. At this stage, implantable BioMEMS devices can provide accurate measurements (SNR > 20 dB) that are not affected by motion, and their embedded firmware allows for real‐time validation of the data and secure sending [181]. Because of this hardware‐software partnership, data integrity and security from the sensors to higher levels can be met. The decision‐support layer combines BioMEMS and wearable data to help resolve any conflicts between the sensors [182]. Data visualization approaches and similar tools in TAI reveal the role of each biosensor in diagnosis, which doctors can easily interpret. At the same time, rules are followed with particular protocols (IEEE 11073) for firmware updates and data processing, which have led to a significant improvement in equal care delivery. It links the latest technology with the safety and reliability needed in telehealth [183, 184].

### 6.3 Wearable Health Monitors

Wearable devices integrate BioMEMS for non‐invasive measurement of key health indicators, such as HRV, hydration status, body temperature, bladder pressure, and sometimes stress levels via skin conductance or ECG [185]. These wearable devices, when fed into telehealth platforms, produce a steady stream of patient data for healthcare providers to monitor and adjust a treatment plan in real‐time for each patient [186]. For example, the Eversense CGM system from Senseonics enables patients to get real‐time glucose readings through its implantable sensor, which functions for 90 days at a time. The technology finds practical use in telehealth programs that assist remote diabetes management for patients with type 1 diabetes. Various clinical studies document how the Eversense system enhances blood sugar management while decreasing occurrences of low blood glucose incidents [187].

### 6.4 LOC Diagnostics in Telehealth

A specialized form of BioMEMS, LOC systems are miniaturized laboratories on a single chip capable of complex biochemical tests (e.g., blood or urine analysis) [188]. By being small, portable diagnostic tools, they provide a perfect solution for remote diagnostics, reducing the need for visits to the lab, which further increases healthcare accessibility, particularly in remote or less well‐served regions. In addition, they can analyse blood or urine in real‐time and can be incorporated into telehealth platforms, unlike conventional electrodes that cannot perform this diagnostic and lab‐level functionality. This allows for highly tailored healthcare via real‐time, biochemically rich input data, better than the simpler metrics provided by conventional PPG or ECG electrodes [189].

### 6.5 Drug Delivery Systems in Telehealth

Implantable drug delivery systems that indeed release specific doses of medication at pre‐determined times are also a form of BioMEMS technology [190]. These systems, which can be programmed remotely, are used by healthcare providers to control conditions such as pain, diabetes, cancer, and hormonal disorders from a distance. Clinicians can adjust medication dosages in real‐time through a telehealth connection so that patients don't need to travel to healthcare facilities for personalized, precise care. BioMEMS ultimately offers a complete telehealth care approach that enables personalized treatment, increases access, and decreases the healthcare burden by reducing the need for in‐person visits [191]. For example, implantable drug delivery systems, such as the Medtronic SynchroMed II pump, have been deployed in telehealth programs for the management of chronic pain and spasticity [192]. The compact and efficient design makes them attractive for continuous health monitoring and patient‐specific interventions in telehealth environments.

## 7 Technical Challenges and Risks in Telehealth

In the context of telehealth, there are many technical challenges and risks related to firmware design and development, security, noise and interference management, intrusion prevention, and adaptability to extreme environmental conditions [193]. However, each of these elements is crucial to ensuring the reliability, accuracy, and security of telehealth systems.

### 7.1 The Critical Role of Firmware in Telehealth

Firmware is the middleman between hardware and higher‐level applications that are used to control, manage, and support sensors and monitoring systems. Firmware design and development are of utmost importance in telehealth systems because they guarantee that data acquired from sensors is transmitted to the system without error and continuously [194]. In addition, Firmware allows the system to be easily adaptable to improvements and future technologies. The system must be designed to work effectively under varying environmental conditions and various user needs to ensure reliability and consistency. However, the challenge is implementing firmware, and that takes specialized expertise and significant financial investment [195]. As well as the operational reliability and security performance of AI‐driven telehealth systems depend on firmware, while developers encounter significant challenges in maintaining it [196]. Modular building design in firmware management enables versatile system operation and scalability to perform updates that avoid service interruptions. Over‐the‐air (OTA) automatic updates immediately provide both security updates and performance enhancements to users. The accepted standard IEEE 11073 enables telehealth systems integration because ML uses it to detect system problems before they appear [197]. Open‐source collaboration initiatives combined with regulatory compliance enable the telehealth system to gain trust and improve the reliability of firmware solutions [198]. These strategic approaches resolve firmware problems to enhance the operational performance and access capabilities of AI‐based telehealth systems [199]. The performance quality and security depend heavily on firmware optimization for AI‐driven health devices. The optimization strategies for firmware implementation consist of modular design and OTA updates, with energy efficiency, security protocols, and real‐time processing as outlined in Table 5. The strategies resolve different obstacles to scalability, power management, and data protection as they improve telehealth system performance.

**TABLE 5:** The optimization strategies for firmware in AI‐driven health devices.

**Strategy**	**Description**	**Benefits**	**Challenges**
Modular design	Enables scalable and adaptable firmware updates.	Facilitates easy updates and system scalability.	Requires careful planning and integration.
OTA updates	Allows remote firmware updates for security and performance enhancements.	Reduces downtime and ensures devices are up‐to‐date.	Requires robust network infrastructure and security protocols.
Energy efficiency	Optimizes firmware to reduce power consumption in wearable devices.	Extends battery life and improves device usability.	May require trade‐offs with processing speed or functionality.
Security protocols	Implements encryption and authentication mechanisms to protect sensitive data.	Enhances data security and patient privacy.	Increases computational overhead and complexity.
Real‐time processing	Enhances firmware to support real‐time data analysis and decision‐making.	Enables timely interventions and improves patient outcomes.	Requires high‐performance hardware and efficient algorithms.

### 7.2 Security Challenges in Telehealth

Data security and patient privacy are among the most pressing concerns in telehealth systems. These systems, which transmit sensitive personal and health‐related information, require robust protection to guard against potential threats and unauthorized access [200]. Implementing strong encryption protocols such as blockchain, robust authentication mechanisms, and access controls is an essential security measure. These aspects are especially critical in scenarios where network access may be limited or vulnerable, further emphasizing the need for resilient security frameworks [201]. The secure patient data storage framework of blockchain operates from a decentralized system that keeps data intact through tamper‐proof methods while connecting to the current infrastructure through key components. The distributed nodes of blockchain storage maintain data decentralization, which improves system resilience through encrypted block development that provides authorized access [93]. The system generates unalterable audit trails to document all transactions, which helps organizations meet requirements from HIPAA and the general data protection regulation (GDPR) [202]. Smart contracts, through predefined rules, perform access control by either activating or disabling data access automatically [203]. Also, blockchain technology provides a secure EHR system for data sharing through its integration with telehealth platforms by using APIs and middleware [90]. MIT's MedRec provides a decentralized EHR system, while the fast healthcare interoperability resources (FHIR) chain establishes a combination of blockchain technology with FHIR standards to secure information while enabling better network connectivity [204]. In addition, telehealth systems managed by AI should be integrated with EHRs to allow smooth data sharing as well as better clinical decisions and superior patient results. Obtaining interoperability brings substantial hurdles due to different EHR execution patterns and complicated processes for integrating unstructured data from AI systems. Real‐time data exchange becomes possible due to standardized protocols, FHIR and HL7, together with API and middleware solutions, which create system compatibility [205]. Accurate and uncorrupted data processing occurs through synchronous capability based on real‐time updates and blockchain audit trails. The tools do not resolve ongoing challenges such as data silos and vendor‐specific implementations, which demand continuous teamwork among developers, providers, and vendors [206]. The practical implementation of interoperable healthcare solutions leads to decreased duplicated entry of patient data, along with enhanced care coordination and regulatory compliance practices such as HIPAA and GDPR, among others, which help patients develop trust [207].

### 7.3 Noise and Interference Management

Telehealth systems are frequently deployed in real‐world, noisy environments where various forms of interference can affect sensor data quality [208]. For instance, minor changes in the user's physical state, environmental noise, and electromagnetic interference can distort data accuracy [209]. Therefore, developing advanced algorithms capable of noise reduction and interference mitigation is crucial for enhancing data quality and ensuring the reliability and trustworthiness of telehealth systems [210].

### 7.4 Intrusion Detection and System Safety

Another technical challenge is safeguarding systems against cyber intrusions and attacks. Telehealth systems often operate over public or private networks, making them susceptible to cybersecurity threats [211]. Employing robust firewalls, intrusion detection and prevention systems, and modern cybersecurity technologies can mitigate the risk of intrusion and maintain the integrity of patient data and system functionality [194, 200].

### 7.5 Adaptability to Extreme Environmental Conditions

The performance of telehealth systems in extreme conditions such as very high or low temperatures, high humidity, or unstable atmospheric conditions also poses a considerable challenge. Firmware and hardware in these systems must be designed to withstand such harsh environments to maintain their functionality and accuracy. Using resilient materials and weather‐resistant designs enhances the reliability of these systems in diverse environmental conditions [210].

### 7.6 Cost Implications of AI‐Driven Telehealth Systems

The implementation of AI technology within telehealth systems offers both financial benefits and implementation difficulties regarding cost. AI‐driven solutions lower long‐term healthcare expenses, but significant costs emerge from developing and obtaining data, along with training models during initial implementation. Existing infrastructure integration with AI technology requires substantial spending, since it includes both customization work and solving interoperability problems [212]. The ongoing expenses for maintaining model performance involve updating systems, implementing cybersecurity protocols, and adhering to regulations because these features support data protection and system effectiveness. The implementation of AI‐driven telehealth networks produces cost‐reduction through decreased physical patient examinations and efficient resource handling, which consequently leads to better chronic condition treatment resulting in diminished medical facility re‐admissions [213]. The issue of providing equal healthcare access in limited‐resource areas remains difficult to solve because high expenses can create more health inequality. Open‐source AI models, along with affordable biosensors, can be supported through government subsidies to minimize this issue as part of public‐private partnership initiatives [214]. The profitability of AI telehealth solutions hinges on both the system scale and operational efficiency, but patients and organizations obtain extended value through improved outcomes and system optimization that justifies initial financial commitments. A research study reveals how remote monitoring with the support of AI tools lowered readmissions in health institutions by 20% thus showing the potential for significant financial savings. The achievement of equitable and sustainable AI‐driven telehealth solutions requires proper management of economic factors [215].

### 7.7 Ethical Considerations

Ethical considerations have always been critical to the design and deployment of firmware in telehealth [36]. Because firmware deals closely with sensitive health data and personal information, we also make sure patient privacy, informed consent, and data security are met [216]. To counter concerns of misuse of data or of data being accessed unnaturally, the development process must be held to the highest standards of transparency and accountability [217]. These present an ethical challenge that has to be prepared in advance and backed up by solid safeguards, especially in situations where patients are at risk and potentially critical choices regarding their health are involved [36, 218].

### 7.8 AI Bias and Fairness in Telehealth

The implementation of AI in healthcare demands complete evaluations of bias and fairness to preserve equitable healthcare services [216]. The specific susceptibilities of AI systems with ML capabilities stem from misdirected training samples, incorrect algorithm settings, and insufficient population representation data [212]. Healthcare biases generate unequal patient outcomes, affecting medical decisions for diagnosis, along with treatment choices and care involvement, specifically damaging underprivileged groups. The necessary action plan for managing these concerns includes three main requirements to employ fairness‐aware algorithms, together with diverse dataset development and continuous monitoring systems to evaluate AI solutions [219]. XAI techniques give healthcare providers visibility into AI‐driven recommendations to detect and minimize biases that occur. Telehealth systems equipped with TAI technology will deliver care that focuses on patients through equitable treatment for all patients [220].

### 7.9 Regulatory Challenges in AI‐Driven Telehealth

To successfully implement AI in telehealth, one must address the complicated set of regulations, including HIPAA in the U.S. and the GDPR in the European Union [221]. These standards establish strong requirements for data protection, along with security protocols, together with patient authorizations specifically applied to AI systems that handle vulnerable medical information. Medical platforms in telehealth need to use strong encryption, along with access restrictions and data auditing solutions, to protect personal health records according to HIPAA requirements [222]. Under the GDPR, patients have the right to access their health data, while the regulation also requires complete transparency in data processing activities. AI‐driven telehealth systems need to satisfy the requirements of medical device regulations enforced by the FDA. AI algorithms used in medical diagnostics and therapy need to pass medical device qualification, since they require extensive testing and approval procedures before they can be deployed. AI‐based diagnostic devices need to prove their clinical safety and reliability through FDA clearance by meeting accuracy requirements through clinical trials [223]. The time required, along with the expense for regulatory review, creates a substantial obstacle to prompt telehealth adoption of new AI systems. The absence of consistent regulations regarding AI in healthcare generates procedural ambiguity for those developing healthcare systems and their medical service providers. Different jurisdictions maintain separate regulatory needs, which create challenges when providers attempt to deploy telehealth solutions across international borders. The successful integration of telehealth AI innovations into clinical practice requires stakeholders to partner with regulatory bodies to create standardized guidelines and standards [224].

### 7.10 Real‐World Case Studies of AI‐Related Data Breaches in Healthcare

The essential requirement for AI‐driven telehealth systems to implement strong cybersecurity becomes apparent through current real‐life incidents [225]. A large healthcare organization became a victim of ransomware targeting its AI‐operated patient surveillance platform, which resulted in encrypted sensitive information, together with interrupted service delivery, thus validating encryption measures with emergency response protocols [226]. The patient data leak from an AI diagnostic platform happened because of poor authentication measures, which revealed the essential need for adding multiple authentication factors [227]. A telehealth company experienced internal misuse, which exposed previously unknown security risks because of an employee who became an insider threat; therefore, they required stronger access controls and monitoring systems [228]. The security flaws within wearable device firmware opened a path for hackers to steal current health data, thus proving the importance of firmware protection. Patients require protection from the extreme consequences of encryption access controls and regular updates to maintain trust in telehealth systems because of recent breaches [229].

### 7.11 Mitigating Biases in AI‐Powered Healthcare

The attainment of equal patient medical care depends on resolving bias problems in AI systems employed in healthcare. The implementation of AI systems requires structured datasets that represent various groups of patients to achieve accurate performance for all patient demographics. AI Fairness 360 from IBM and fairness metrics need to be used for regular auditing and bias detection operations [230]. The XAI techniques LIME and SHAP provide medical staff with transparent AI decision‐making, so they can both understand system choices and detect sources of bias. AI recommendations pass through human evaluation systems, which both validate them and improve their performance at each step [231]. The incorporation of adversarial debiasing techniques as algorithmic fairness constraints helps reduce biases that appear during training procedures. These implementation measures lead to delivering equal, high‐quality healthcare services for every patient [232].

## 8 Discussion and Conclusion

**FIGURE 6:**
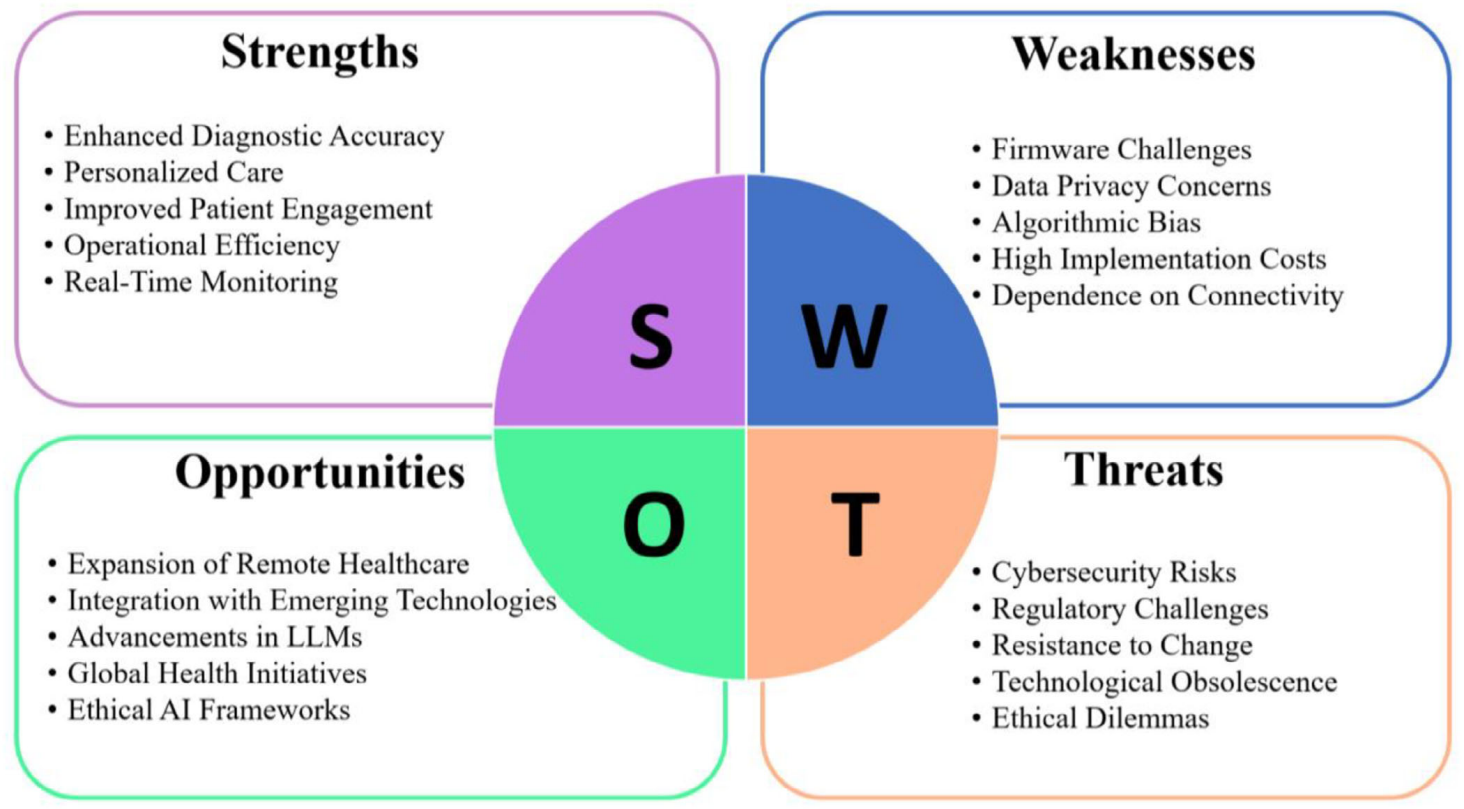
The SWOT analysis in telehealth.

Integration of TAI and knowledge‐based systems into telehealth represents a transformative approach to modern healthcare, which enables continuous, real‐time monitoring and decision support, particularly in remote and underserved environments [233]. These systems leverage advanced LLMs in conjunction with structured knowledge frameworks to introduce a paradigm shift in telehealth with robust data analysis and actionable recommendations derived from complex, high‐frequency data from wearable and implantable biosensors. Such progress transcends those common elements of telemedicine to establish a proactive healthcare environment that fosters personal, tailored patient monitoring for chronic diseases, post‐operative care, and acute situations requiring immediate response. TAI can enhance the precision and reliability of both physiological and psychological health monitoring using wearable biosensors, such as cardiac monitors (such as the Apple watch), blood glucose tracking tattoos, and implantable devices. This capability enables clinicians to evaluate patient data in real‐time, a critical attribute to personalizing care and improving preventive healthcare. AI systems can help clinicians identify health trends early on and flag potential risks, enabling them to take preemptive actions and improve patient outcomes. That's the kind of proactivity that modern healthcare is looking for: moving from reactive care to preventive care and directly impacting patients’ long‐term health trajectories. However, technical challenges exist in implementing TAI‐powered telehealth systems. The key layer that makes the interaction between hardware (e.g., sensors) and software (e.g., analysis algorithms) smooth and accurate is firmware. Given the importance of maintaining data integrity in high‐stakes clinical environments, where interoperability and real‐time response are critical, consistent firmware performance across devices is essential. Data security also depends on firmware reliability, because firmware vulnerabilities can expose sensitive patient data to security breaches. For that reason, the design of robust firmware with security protocols and anti‐tamper measures is fundamental to maintaining patient trust and the operational effectiveness of TAI‐driven healthcare systems. Reliable and trustworthy telehealth systems based on TAI depend on having strong firmware security measures in place. We have found that using well‐constructed cryptographic protocols supports both data security and a responsive system. Such security features on the hardware greatly decrease the risk of data breaches without affecting necessary monitoring activities. In addition, these secure firmware architectures have shown better accuracy in diagnosing critical care patients, and they fulfill strict medical device certification rules. Immutable auditing features handle key regulatory matters, and clinicians point out that strong security is more important than extra functions. This means that firmware plays a key role in bridging the gap between technical capabilities and clinical acceptance, especially for regulated medical devices that must balance security and performance. The deployment of TAI in telehealth also involves considering the important role of ethical considerations. Essential to establishing trust in these systems is transparency in the data usage, patient consent, and an AI‐driven decision‐making process. We must make patients aware of the role they play in adaptive AI models, and the specifics of how their data will be used and protected. Lastly, the transparency of AI algorithms is crucial for clinicians to have faith in how an AI system recommends a course of action. It can be reached through the addition of XAI mechanisms that aid clinicians in understanding and validating the system's recommendations to form a community of trust and collaboration between technology and healthcare providers. Telehealth combines DL algorithms, including CNNs and transformer models, which specifically include LLaMA and ChatGPT, to enhance diagnostic imaging and predictive modeling and NLP functions that demonstrate equal or superior performance to human experts [234]. The tools operate with high‐quality data while facing transparency issues, but provide quick, accurate analysis and support clinical decision‐making, thereby reducing healthcare loads. The application of AI technology delivers the most value to remote locations because it enhances medical effectiveness while streamlining healthcare operations [114]. A major challenge here is to combat algorithmic biases that otherwise could lead the system's recommendations to be not only wrong but also unfair. TAI‐based telehealth applications potentially affect critical patient outcomes and therefore need rigorous ethical standards that reduce bias by providing for data use, fairness, and inclusivity in algorithm development. Such systems should have integral bias detection and correction mechanisms; these mechanisms will determine both the quality of care and how well all patients are treated equally. To overcome these technical, ethical, and operational challenges, TAI‐based telehealth systems must be rigorously piloted in controlled, context‐specific environments. Pilot studies allow researchers and engineers to evaluate possible bottlenecks and security problems and hear ethical implications, which are very beneficial for the refinement of the system's design. These trials should include contributions from AI developers, healthcare providers, and legal experts to thoroughly evaluate those complexities that are realized in our real world. Pilot programs involve simulating as diverse clinical scenarios as possible to identify and mitigate firmware inconsistencies, ethics issues, and smooth integration before applying the full scale. For better evaluation, the strengths, weaknesses, opportunities, threats (SWOT) analysis [235] is shown in Figure 6.

Also, the implementation of TAI in telehealth produces various technical and ethical problems as well as regulatory and operational boundaries. The successful implementation of TAI telehealth depends on two things: optimizing the firmware to ensure reliable data integrity and system performance while establishing strong data protection measures, reducing noise levels, and promoting cross‐data source connectivity [236]. The deployment of AI‐driven recommendations requires resolving ethical issues involving algorithmic discrimination and the requirement of transparency and patient consent provisions for establishing AI fairness alongside human‐centered care systems to resist their dehumanizing potential [118]. The absence of standardized frameworks, together with high implementation costs, prevents scalability most strongly in low‐resource settings because of regulatory hurdles. The reluctance to embrace change, along with a dependency on secure internet connectivity, restricts remote areas from adopting TAI solutions [237]. Studies encounter three main limitations because they lack a broad application of controlled research results, mostly concentrating on short‐term observations, and need high‐quality data to achieve optimal AI performance [238]. Furthermore, the limited data availability prevents AI from accessing diverse high‐quality datasets and merging biosensor and EHR data successfully. Research data shows AI biases start to form during data imbalance phases of algorithm programming, while these factors together intensify healthcare disparities that particularly affect minority populations. SHAP, together with LIME, serves as two primary XAI methods that collaborate with Fairness‐aware algorithms to decrease biases, along with enhancing system readability. Research must evaluate ethical issues together with data protection rules, and HIPAA/GDPR to help healthcare organizations deploy AI solutions across various regions. The deployment of comprehensive and reliable AI‐based telehealth systems across different areas depends on solving existing obstacles first. In conclusion, AI‐driven telehealth system success depends on active collaboration between three main stakeholders, including patients, clinicians, and AI developers. Patients need to give feedback about user experience to ensure AI tools provide accessible interfaces that meet their requirements [239]. Patient involvement during design tests helps detect usability problems while establishing their trust in AI systems. Clinicians need to participate throughout technology development to guarantee AI tools improve clinical operations rather than cause disruptions [240]. The integration of AI recommendations into decision systems requires the valuable expertise of clinical staff members for validation and implementation into organizational processes. AI developers should establish complete transparency while keeping ethical components and explainable functionality their top priorities during development periods by engaging extensively with both medical staff and patients to solve privacy and data protection issues, alongside bias‐related matters. Support from regulatory bodies, policymakers, and interdisciplinary group work, enables the creation of advanced telehealth systems powered by AI, which combine equality with patient focus and efficient clinical care. Stakeholder cooperation based on their objectives and knowledge will establish an optimized environment where AI can effectively transform health services [241].

### 8.1 Roadmap for Implementing TAI in Telehealth

A systematic framework is required to integrate TAI into telehealth systems properly. Below is a seven‐step framework:

Step 1. The setting of ethical regulations and regulatory frameworks must include standards for transparency, together with fairness and accountability measures that meet the requirements from HIPAA and GDPR.
Step 2. Healthcare groups should build an extensive data governance framework that includes both quality, privacy, and security protocols for protected patient information.
Step 3. XAI methods, including SHAP and LIME, should be integrated to enhance understanding of AI‐driven recommendations and establish trust in AI systems.
Step 4. The system should maintain secure data processing by using a modular design and OTA updates in combination with energy‐efficient firmware.
Step 5. AI systems need pilot tests that run performance evaluations to handle ethical problems and improve algorithms through feedback from the medical environment.
Step 6. Healthcare providers and patients, together with policymakers, should collaborate to guarantee that AI systems fulfill clinical and ethical requirements.
Step 7. The evaluation of AI systems concerning compliance issues, as well as performance standards and bias detection, needs to be performed continuously to maintain long‐term operational integrity and dependability.

The roadmap creates an organized system for stakeholders to integrate TAI in telehealth to achieve secure and equitable medical care.

### 8.2 Future Directions

The upcoming 5–10‐year period will introduce revolutionary transformations to AI‐based telehealth services that will change medical diagnostics, as well as individualized healthcare and tele‐monitoring systems, and regulatory authorities. Through AI, medical facilities will achieve real‐time, accurate image assessments, including X‐rays and magnetic resonance images (MRIs), matching the ability of specialist medical professionals, thus shortening diagnostic periods and enhancing therapeutic success. Alternative medicine will apply AI processing to genetics, together with environmental and lifestyle factors, to optimize treatment solutions for patients suffering from diabetes and high blood pressure. The future of wearable biosensors involves constant health metric assessment through containment methods, which AI technology will use to identify problems early and prevent them. The approval bodies will develop ethical rules that address the protection of personal data, while also covering biases and privacy standards for making AI systems transparent and unbiased. Standardized protocols such as FHIR and HL7 facilitate smooth data exchange, which lets the AI system receive entire patient records for precise predictions and scalability across nations. LLMs, along with generative AI systems, will establish essential roles in improving clinical support functions, medical report creation, and patient‐practitioner communication using natural language processing capabilities. AI‐powered telehealth services will create an environment that provides exact and personalized healthcare to patients. Finally, this research demonstrates the great promise of TAI‐driven telehealth systems to transform healthcare delivery by enabling precise, proactive care tailored to each patient. These systems can allow healthcare professionals to leverage timely, data‐driven insights to improve patient outcomes and build trust in AI‐powered healthcare solutions by optimizing firmware, developing robust security frameworks, and ensuring algorithm transparency. While we are moving toward broader adoption, it will be critical to have a balanced focus on performance, security, and ethical integrity to realize the transformative potential of TAI in healthcare fully. This systematic review identified several challenges and limitations that may affect the comprehensiveness and accuracy of literature evaluation. Despite double‐checking measures, relevant studies may have been missed through the use of keyword constraints or sorting criteria in major databases (e.g., IEEE Xplore, PubMed), and a range of search terms to enhance construct validity. Errors from the large workload in data extraction, comparison, and coding were mitigated with cross‐checking, but some misinterpretations may remain. In addition, our findings are limited in their external validity due to limitations in the generalizability of our results to other telehealth contexts and methods. Future systematic reviews of telehealth would achieve improved consistency, reproducibility, and applicability if they used standardized frameworks with broader study scopes.

### 8.3 Conclusion

This review identified how TAI, together with LLMs, wearable biosensors, and knowledge‐based systems, affects telehealth platform implementation and ethics during operations. Research demonstrates that TAI enhances clinical decision support, patient interaction, and operational efficiency through LLaMA and ChatGPT, along with other LLMs, which boost symptom understanding and medical diagnosis. Biosensors attached to the body, together with BioMEMS, provide continuous patient monitoring to generate individual treatment recommendations. The system reliability depends on resolving three critical issues, including firmware optimization and data security, as well as noise management. The implementation of TAI creates ethical matters because it exposes patients to algorithmic biases and privacy risks, which need XAI systems and fair algorithms to combat these problems. TAI delivers better diagnostic results, patient outcomes, and resource efficiency than traditional telehealth, yet needs proper deployment to manage associated safety concerns. Local experts from both engineering fields, medical branches, and policy groups need to form teams to overcome TAI implementation challenges. Future research needs to validate TAI clinical outcomes over extended periods while creating standardized procedures for data protection and both technical and moral compliance. The resolution of these gaps will allow TAI to transform telehealth into an improved system that offers fair patient care and enhances operational efficiency. Telehealth receives a revolutionary healthcare upgrade through TAI and knowledge‐based systems that support real‐time patient checks, personalized medicine delivery, and clinical decision systems for areas that lack medical support. System architectures that combine LLMs and biosensor data enable healthcare providers to generate valuable discoveries that help manage chronic diseases and understand post‐operative recovery and emergencies. Their achievement depends on resolving multiple essential obstacles. The smooth functioning between hardware and software components relies on optimized firmware because it enables data consistency and system connection while delivering instant responses. The protection of sensitive patient information has become a primary priority, so healthcare providers require highly advanced encryption measures, as well as intrusion detection systems and full cybersecurity frameworks. Scalability and reliability face obstacles because of the need to deal with noise interference, harsh environmental conditions, and the high costs of AI implementation. Ethical considerations are equally vital. Satellite health services must ensure clear data practices, combined with patient‐consent protocols and easy‐to‐understand AI decision processes, for healthcare providers to trust the system. Equitable healthcare delivery depends on two critical measures: first, we need to fix algorithmic bias; second, we need to maintain fair treatment across different patient ethnic groups. AI development needs ethical guidelines that establish responsibility and comprehensive inclusion, especially in situations with high risk, such as emergency medical situations. The success of AI‐driven telehealth in the future requires the combined solutions of extensive testing, cross‐sector partnerships, and prolonged assessments of the system. TAI systems will provide precise, patient‐centered, proactive care when firmware optimization, security improvement, and algorithm transparency measures are implemented. The combination of LLMs with knowledge‐based systems will enhance both clinical decision quality and patient participation in healthcare services. The deployment of sustainable and fair telehealth solutions needs technical excellence to align with ethical standards that ensure patient well‐being through trust and safety protection for all users.

## Author Contributions

J.G. and F.S.K. were responsible for conceptualizing the study framework and designing the research methodology. F.S.K. supervised the project, oversaw data acquisition, and provided critical insights that shaped the study's direction. J.G. and F.S.K. conducted data analysis and performed statistical evaluations to ensure accuracy and rigor in the findings. S.A.Z. led the manuscript drafting and initial writing, while all authors, including J.G., F.S.K., and S.A.Z., contributed to the manuscript's revisions, ensuring it met intellectual standards. All authors have reviewed and approved the final version of the manuscript for submission.

## Ethics Statement

The authors have nothing to report

## Consent

All authors have thoroughly reviewed and approved the final manuscript for publication. They have given their consent for its submission to the journal and agree to its publication in its present format.

## Conflicts of Interest

The authors declare no conflicts of interest.

## Data Availability

Data sharing does not apply to this article as no datasets were generated or analysed during this systematic review. All data analysed were derived from published articles retrieved from reputable online databases, including Springer, ScienceDirect, Elsevier, IEEE, PubMed, and Emerald Insight.
